# Squamous Cell Carcinoma of the Sigmoid Colon: A Path Less Traveled

**DOI:** 10.7759/cureus.22297

**Published:** 2022-02-16

**Authors:** Deepti Ramachandra, Gourav Kaushal, Anvin Mathew, Puneet Dhar, Nirjhar Raj Rakesh

**Affiliations:** 1 Surgical Gastroenterology, All India Institute of Medical Sciences, Rishikesh, IND; 2 Surgical Gastroenterology, All India Institute of Medical Sciences, Bathinda, IND

**Keywords:** bladder cancer, metachronous cancer, second primary, squamous variant, colon cancer

## Abstract

Squamous cell carcinoma (SCC) involving the gastrointestinal tract is exceptionally rare, except in the esophagus and the anal canal. In the hindgut, a common site of involvement is the colo-rectum, commonly seen in the fifth decade of life. The presentation is usually in the advanced stages and carries a poor prognosis. Due to the rarity of the disease, before labeling it as a primary lesion, the possibility of metastasis from a distant primary should be entertained. Consensus guidelines regarding the management of such a rare condition are lacking. Here, we present the case of an elderly gentleman with a history of surgery for urinary bladder cancer 20 years back (the nature of which is not known). The patient presented with left lower abdominal pain and altered bowel habits. His pain had persisted for approximately two months along with a recent onset of overflow incontinence but no other associated constitutional symptoms. Examination revealed pallor and a vague abdominal mass in the left iliac fossa. On further evaluation with a colonoscopy, a growth was seen in the sigmoid colon. Computed tomography of the abdomen revealed a locally invasive growth arising from the sigmoid colon along with a space-occupying lesion in the left lobe of the liver enhancing on the portal phase. Biopsy from the sigmoid and the liver lesion was reported as SCC which was confirmed by immunohistochemistry. Given the metastatic nature of the lesion, treatment options were discussed in a multidisciplinary team setting, and the decision was made to proceed with diversion colostomy and palliative chemotherapy. SCC of the colon is a rare disease and is usually diagnosed at an advanced stage. Even in operable cases, the prognosis is dismal, and various treatment modalities have been attempted. Due to the rarity of the disease and paucity of data regarding definitive management, treatment varies from one patient to another.

## Introduction

Primary squamous cell carcinoma (SCC) of the gastrointestinal tract (GIT) is rare, except in the esophagus and anal canal. The most common location of SCC in the hindgut is the colo-rectum [1]. It accounts for approximately 0.1-0.25% per 1,000 diagnosed colorectal carcinomas [2]. In the early 20th century, Schmidtmann reported SCC of the caecum, and Raiford published SCC of the rectum [3]. Most cases have been reported in the rectum and appear to affect both genders, but colonic lesions appear to have a male preponderance and are thought to present by the fifth decade of life [4,5]. Presentation is usually in advanced stages and carries a dismal prognosis [5]. Metastasis from a distant primary should be entertained before labeling it as a primary lesion. Metastasis is usually to the small bowel, especially from the lungs. As the disease is rare, definite consensus regarding the management is lacking. Here, we present the case of a patient who developed a second primary (colonic SCC) after undergoing radical cystectomy and an ileal conduit for urinary bladder carcinoma.

## Case presentation

**Figure 1:**
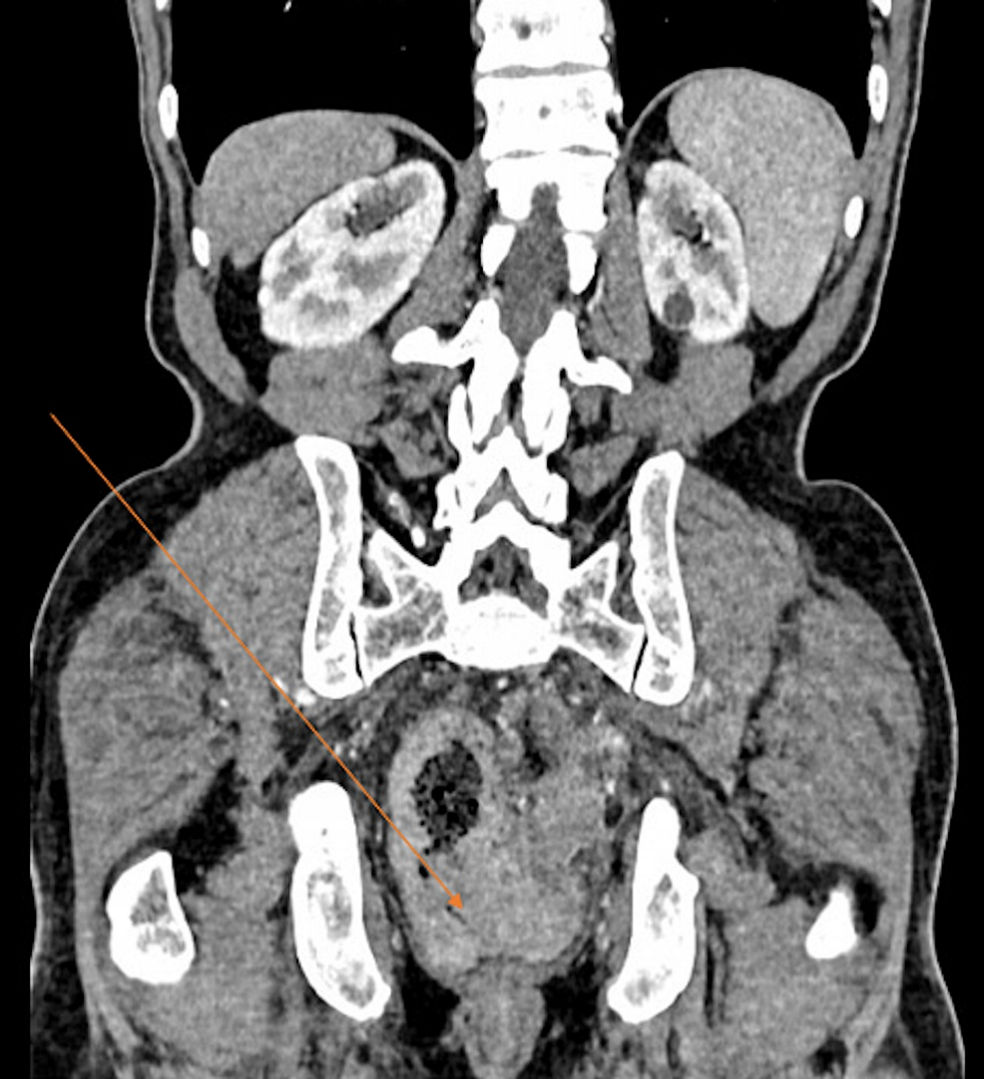
Coronal section of contrast CT scan of the abdomen. The arrow denotes circumferential thickening of the segment of the sigmoid colon causing a stricture and upstream dilatation of the loop of the colon. The lesion extends beyond the serosa and invades the adjacent rectum.

**Figure 2:**
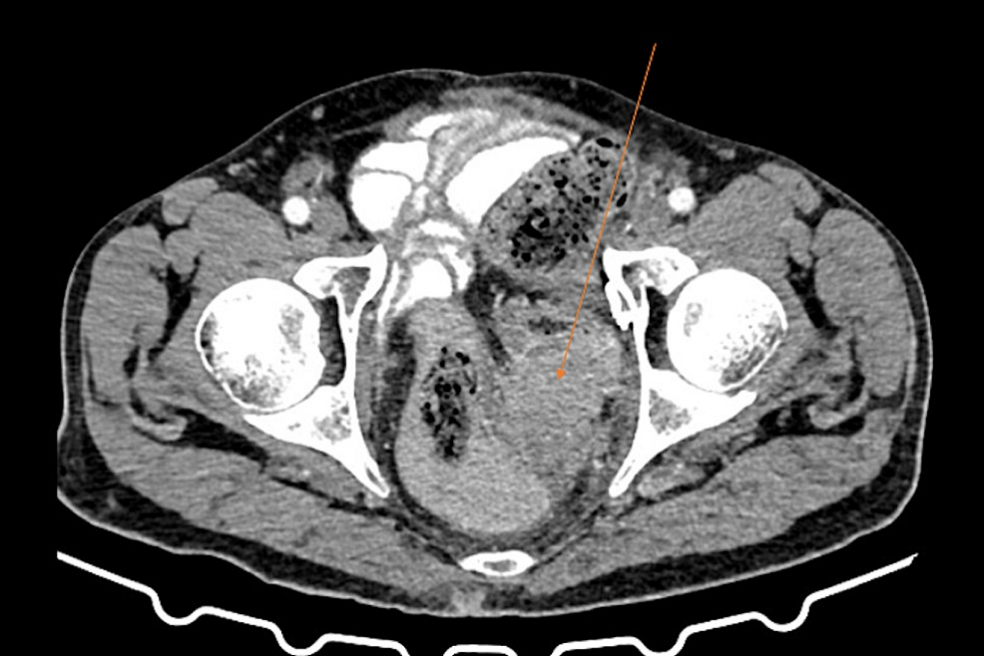
Axial section of contrast CT of the abdomen. The arrow shows a mass lesion in the sigmoid colon and involves the left obturator space.

**Figure 3:**
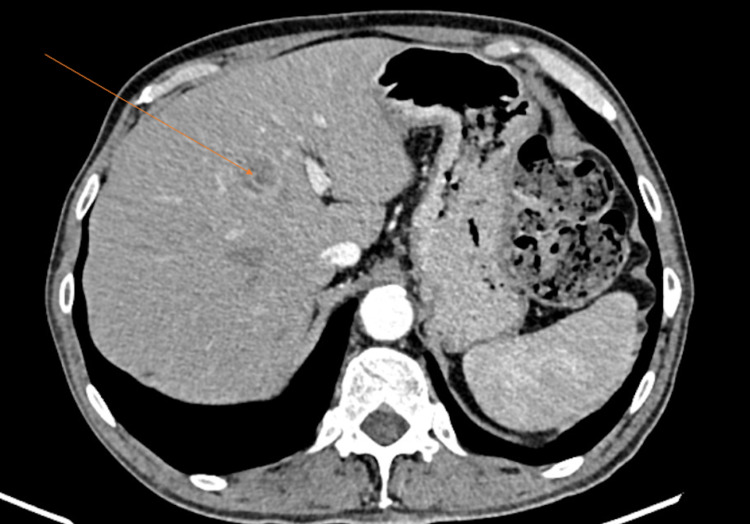
Axial section of triphasic CT of the abdomen. The arrow depicts a hypoenhancing, space-occupying lesion in the segment IV of the liver with peripheral enhancement on the portal phase.

**Figure 4:**
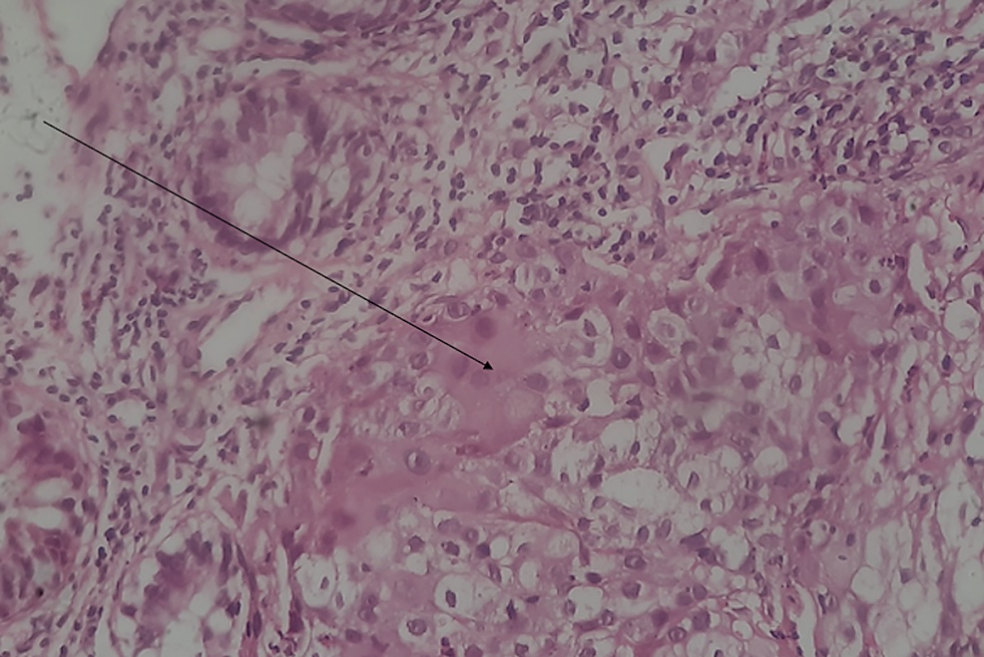
Histopathology slide of the colonoscopic biopsy specimen. The arrow shows sheets of polygonal cells with eosinophilic cytoplasm.

**Figure 5:**
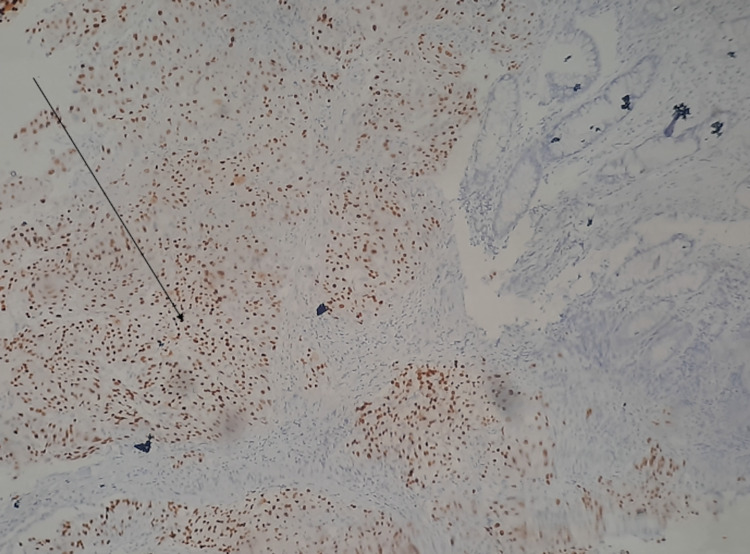
Immunohistochemistry of the colonoscopic biopsy specimen. The arrow shows positively stained (brown) P40-expressing cells.

**Figure 6:**
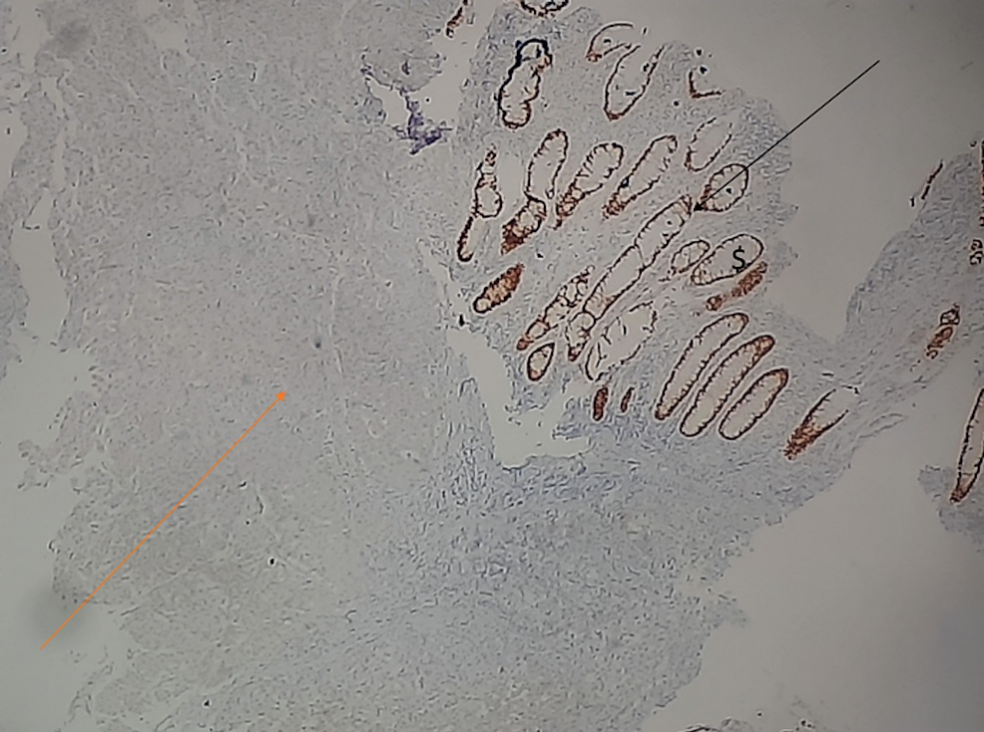
Immunohistochemistry of the colonoscopy biopsy specimen. The black arrow depicts normal colonic glandular tissue positive for CK-20, and the orange arrow depicts tumor tissue negative for CDX2 and CK-20-expressing cells. CK: cytokeratin; CDX2: caudal-type homeobox 2

Our patient was an elderly gentleman with no medical history but a surgical history of radical cystectomy and an ileal conduit formation two decades ago for urinary bladder malignancy. The nature of the urinary bladder lesion could not be ascertained due to a lack of records. The patient presented with pain in the left lower abdomen and altered bowel habits for two months along with symptoms of overflow incontinence for a week. He had no anorexia, weight loss, or any rectal symptoms. He provided a history of tobacco use in the form of 20 beedis per day but had quit about 25 years ago. In addition, he provided a history of occasional alcohol consumption before the diagnosis of urinary bladder cancer and had quit ever since. His family history was non-contributory. On examination, he was a thinly built man with a body mass index of 21.6 kg/m2 and a good performance score (World Health Organization score 1). He had mild pallor, and the rest of the general examination was non-contributory. Abdominal examination revealed a well-healed midline laparotomy scar with a healthy urostomy in the right lumbar region. A vague mass was palpable in the left iliac fossa of the abdomen. On evaluation with basic blood parameters, he was found to have anemia and hypoalbuminemia (Table 1). On evaluation with colonoscopy, an ulceroproliferative lesion was seen in the sigmoid colon approximately 35 cm from the anal verge. Further evaluation with computed tomography (CT) showed that the lesion was invading the serosa, the obturator canal, and the rectum, along with a space-occupying lesion (SOL) in segment IV of the liver, which was enhancing on the portal phase (Figures 1, 2). Contrast CT of the thorax ruled out metastasis to the lung. The lesion in the sigmoid was biopsied during colonoscopy, and a percutaneous core biopsy of the liver SOL was performed. Histopathological examination from the lesions revealed features of SCC (Figure 3) which was confirmed on immunohistochemistry (P40+, negative for caudal-type homeobox 2, and cytokeratin 20) (Figures 4-6). After a multidisciplinary team discussion, in view of the advanced nature of the lesion with obstructive features and failed colonoscopy stenting, the patient underwent diversion sigmoid colostomy. Postoperative recovery was uneventful, and he was discharged home on postoperative day five. Chemotherapy with paclitaxel and carboplatin was resumed for palliation. At the six-month follow-up, the patient was well, had gained weight, and had partial tumor response.

**Table 1:** Basic blood investigations, imaging, colonoscopy, and histopathology findings.

Investigation	Results	Normal range
Hemoglobin	10.5 g/dL	13–17 g/dL
Blood urea	30 mg/dL	17–50 mg/dL
Creatinine	0.89 mg/dL	0.7–1.2 mg/dL
Total bilirubin	0.7 mg/dL	0.3–1.2 mg/dL
Direct bilirubin	0.2 mg/dL	0–0.2 mg/dL
Aspartate aminotransferase	29 U/L	0–50 U/L
Alkaline aminotransferase	43 U/L	0–50 U/L
Alkaline phosphatase	110 U/L	30–120 U/L
Albumin	3.1 g/dL	3.5–5.2 g/dL
Prostate-specific antigen	0.04 ng/mL	0–4 ng/mL
Carcinoembryonic antigen	1.7 ng/mL	0–2.5 ng/dL
Colonoscopy	There was an ulceroproliferative growth in the sigmoid colon approximately 35 cm from the anal verge, and the scope was non-negotiable beyond the lesion	-
Histopathology	Infiltration of the tumor with polygonal cells having moderate eosinophilic to clear cytoplasm and hyperchromatic nuclei and nucleoli, suggestive of squamous cell carcinoma	-
Immunohistochemistry on tissue block	Positive for P40, and negative for cytokeratin 20 and caudal-type homeobox 2	-
Triphasic contrast CT scan of the abdomen	Large ulcerated mass lesion approximately 5.3 × 4.4 × 7.9 cm in size noted in the proximal sigmoid colon, causing a stricture measuring approximately 10 × 11 cm and locally invading the serosa and involving the adjacent obturator space and obstructed bowel colon with feral loading. In addition, a hypoenhancing tumor approximately 2.4 × 2.5 cm in size noted in segment IVA of the liver showing peripheral enhancement in the portal phase	-
Contrast CT chest	Calcified nodules in the upper zone and a subcentimetric left subpleural lesion. No lesions were suggestive of malignancy	-

## Discussion

Various reports mention equal preponderance in both genders. It usually affects quinquagenarians [6]. Some of the well-known associations are prior chemoradiotherapy for pelvic malignancies, human papilloma infections, human immunodeficiency virus infections, schistosomiasis and inflammatory bowel disease, fistulous communication with squamous epithelial surface or SCC of the urinary bladder, and duplication of the colon are perceived risk factors. Regrettably, the disease often presents late, and patients have advanced disease while seeking medical attention [5]. It is hypothesized to have a bleak prognosis, probably owing to the diagnostic dilemma and the advanced stage of presentation [7].

It is crucial to consider diagnostic criteria before labeling the lesion as primary colonic SCC. Miyamoto et al. proposed the following four criteria that must be fulfilled to call it a primary SCC carcinoma of the colon [8]: (i) proximal extension from the anal canal should be excluded; (ii) fistulous tract between the squamous lining and involved bowel should be ruled out; (iii) metastasis from other squamous primaries should be ruled out (especially lung); and (iv) histopathological evidence of SCC.

Immunohistochemistry aids in confirming SCC using markers such as P40, CK-AE1/AE3, 34BE12, CK-5, and involucrin, as well as differentiating it from other undifferentiated small cell tumors [9].

SCC of the colon tends to be more locally advanced, with positive regional lymph nodes more often compared to adenocarcinoma [8]. Similarly, the patient had a T4bN1M1 lesion in our case and underwent diversion sigmoid colostomy to relieve the obstruction.

Some of the proposed poor prognostic indicators are poorly differentiated histology, lymph nodal involvement, and ulcerative lesion localized to the left side of the colon. Zhao et al. reported that advanced SCC (stage III and IV) of the colon has a poorer prognosis than adenocarcinoma [10]. The decision of the multidisciplinary team to initiate treatment based on the stage of the disease is pivotal due to the lack of management guidelines. If the epicenter is above the peritoneal reflection, the tumor is treated like a colonic adenocarcinoma, and upfront resection followed by adjuvant chemotherapy is performed [5,8]. Tumors with epicenter below peritoneal reflection are treated like rectal adenocarcinoma, and neoadjuvant chemoradiotherapy is preferred to downsize and downstage the tumor [5]. Because SCC of the colon has a poor prognosis but good performance status, chemotherapy seems reasonable in the palliative setting.

Cisplatin and 5-fluorouracil are the cornerstones of chemotherapy. In addition, various chemotherapeutic drugs such as cisplatin, etoposide, 5-fluorouracil, and etoposide have been used [6,11]. However, the role of radiotherapy is not clear. Generally, radiotherapy in colonic malignancy is ill-advised for fear of radiation enteritis. However, in exceptional cases, such as T4b lesions, it is advocated if deemed resectable and with postoperative positive margins. Colonic stenting is used in obstruction, acts as a bridge to surgery, and can potentially avoid stoma creation [12]. In our patient, colonic stenting was attempted but failed; hence, he had to undergo a diversion sigmoid colostomy.

Our patient developed SCC of the sigmoid colon as a second primary. The first primary was urinary bladder carcinoma about 20 years back. According to a Korean cohort study, although second primary cancers are a common entity in survivors of urinary bladder malignancy, they were generally seen in patients who developed urinary bladder cancer before 50 years of age [13]. The most common second primaries in bladder cancer survivors were prostate, kidney, and lung cancer (both adeno and squamous type), while the incidence of carcinoma of the stomach, liver, colon, tonsil, tongue, and non-Hodgkin’s lymphoma were reduced, as reported by Kwon et al. [13]. Decreased incidence of the risk of the second primary cancer in this particular population of patients is attributed to smoking and alcohol cessation, lifestyle changes, implementing a healthy diet, and exercise [14]. P40 is a consistently expressed isoform in most SCC and has the highest sensitivity and specificity in lung SCC. Because lung pathology was ruled out in our patient, the final diagnosis was colonic primary SCC.

## Conclusions

SCC of the colon is rare and a distinct entity. Therefore, multidisciplinary team effort is desirable in aiding early diagnosis and treatment. As a rule of thumb, metastasis from the squamous primary of the lung should be ruled out, and risk factors such as prior malignancy and chemoradiotherapy are considered. In addition, the idea of a second primary should be cautiously weighed after ruling out metastasis. In resectable colonic SCC, surgery followed by adjuvant chemotherapy is the treatment of choice, while patients with advanced/metastatic stages receive palliative chemotherapy.
